# Pan-Cancer Analysis and Validation Reveals that D-Dimer-Related Genes are Prognostic and Downregulate CD8^+^ T Cells *via* TGF-Beta Signaling in Gastric Cancer

**DOI:** 10.3389/fmolb.2022.790706

**Published:** 2022-02-22

**Authors:** Yiming Guan, Bing Xu, Yi Sui, Zhezhou Chen, Yu Luan, Yan Jiang, Lijuan Wei, Wenjing Long, Sansan Zhao, Lei Han, Dakang Xu, Lin Lin, Qi Guan

**Affiliations:** ^1^ Department of Laboratory Medicine, Ruijin Hospital, School of Medicine, Shanghai Jiao Tong University, Shanghai, China; ^2^ Department of Neurology, Shenyang First People's Hospital (Shenyang Brain Hospital), Shenyang Medical College, Shenyang, China; ^3^ Department of Laboratory Medicine, Shenyang First People's Hospital (Shenyang Brain Hospital), Shenyang Medical College, Shenyang, China; ^4^ Centre for Cancer Molecular Diagnosis, Tianjin Medical University Cancer Institute and Hospital, National Clinical Research Center for Cancer, Tianjin, China

**Keywords:** d-dimer, gastric cancer, prognosis, pan-cancer analysis, TGF-beta signaling, CD8^+^ T cells, coagulation factor, fibrinogen alpha chain (FGA)

## Abstract

**Background:** Cancer is considered one of the most lethal diseases worldwide. Venous thromboembolism (VTE) is the second leading cause of death in cancer patients. As one of the most reproducible predictors of thromboembolism, the D-dimer level is commonly considered by oncologists. Previous studies have demonstrated that the most correlated genes at the D-dimer level are F3, F5 and FGA.

**Methods:** Using data from TCGA and multiple webtools, including GEPIA2, UALCAN, TIMER2.0, Kaplan-Meier Plotter and CIBERSORTx, we analyzed the tumor mutation burden (TMB), microsatellite instability (MSI) and functions of D-dimer-related genes in cancer. Validation was conducted via quantitative real-time polymerase chain reaction (qRT-PCR) and independent GEO + GTEx cohort. All statistical analyses were performed in R software and GraphPad Prism 9.

**Results:** F3, F5 and FGA were expressed differently in multiple cancer types. TMB, MSI and anti-PD1/PDL1 therapy responses were correlated with D-dimer-related gene expression. D-Dimer-related genes expression affect the survival of cancer patients. F3 and F5 functioned in TGF-beta signaling. F3 and F5 were related to immunity and affected the fraction of CD8^+^ T cells by upregulating the TGF-beta signaling pathway, forming an F3, F5/TGF-beta signaling/CD8+ T cell axis.

**Conclusion:** F3, F5 and FGA serve as satisfactory GC multibiomarkers and potentially influence the immune microenvironment and survival of cancer patients by influencing TGF-beta signaling.

## Introduction

Cancer is considered a fatal disease worldwide. In 2020, approximately 1,806,590 new cancer cases and 606,520 cancer deaths were expected in the United States (Siegel et al., 2020). Although millions have died because of cancer, only a few of the cancer types are curable. Traditional cancer therapies rely on surgery, chemotherapy and radiotherapy. However, these treatment methods have not provided a satisfactory prognosis for cancer patients despite their tremendous side effects. Recently, new cancer treatment methods have emerged under the guidance of precision medicine. Antitumor-targeted drugs and molecular diagnosis are typical examples of these attempts at novel treatment. Drugs that target immune checkpoints, such as CTLA-4 and PD1/PDL1, have been used to treat multiple cancer types (Yi et al., 2018; Han et al., 2020). Previously discovered biomarkers have helped medical oncologists. Further innovations towards molecular biomarkers are expected to improve the prognosis of cancer patients.

D-Dimer is a fibrin degradation product comprising a small protein fragment in blood after blood clots are dissolved and degraded by plasmin (Weitz et al., 2017). It contains two D fragments of fibrin, which are linked by crosslinking. D-Dimer elevation in plasma is commonly used in hospitals worldwide to suggest multiple diseases, including venous thromboembolism (VTE), disseminated intravascular coagulation (DIC), pulmonary diseases, diabetes, infection and tumors (Halaby et al., 2015). Its special correlation with venous thromboembolism indicates that D-dimer levels may suggest the survival of cancer patients. Because of its unique features, D-dimer monitoring has been used in patients with geriatric diseases. Previous studies have concluded that particular genes are predictors of fibrin D-dimer levels. A study in 2011 pointed out that three genes—F3 (tissue factor), F5 (coagulation factor V) and FGA (fibrinogen alpha chain)—are associated with fibrin D-dimer levels, in which the association of F3 is the strongest (Smith et al., 2011). As few studies have been conducted to investigate the value of the D-dimer marker at the genetic level; further studies on this topic will help identify new applications of D-dimer.

F3, or tissue factor, is a cell surface glycoprotein (Hisada and Mackman, 2019). This factor enables cells to initiate the coagulation cascade and acts as a high affinity receptor for coagulation factor VII. The resulting complex provides a catalytic event responsible for initiating the coagulation protease cascade through specific limited protein hydrolysis. Unlike other cofactors of these protease cascades, this factor is an effective initiator with complete function when expressed on the cell surface. Tissue factor is considered a link between inflammation and coagulation and has regulatory functions in cancers such as pancreatic cancer and ovarian cancer (Khorana et al., 2007; Cohen et al., 2017; Date et al., 2017).

F5, or coagulation factor V, is an essential cofactor in the blood coagulation cascade (Duga et al., 2004). This factor circulates in the plasma and is converted to the active form by thrombin, releasing the activating peptide during coagulation. This activity creates a heavy chain and a light chain, which are linked together by calcium ions. Activated protein is a cofactor that activates prothrombin to thrombin together with activated coagulation factor X (Segers et al., 2007). Coagulation factor V has been identified as a marker for cancers such as breast cancer (Tinholt et al., 2018).

FGA, or fibrinogen alpha chain, is the alpha component of fibrinogen (Mosesson, 2005). Fibrinogen is a blood-derived glycoprotein comprising three different polypeptide chains. After vascular injury, fibrinogen is decomposed by thrombin to form fibrin, which is the most abundant component of the blood clot. Additionally, fibrinogen and its various lysates regulate cell adhesion and diffusion, show vasoconstriction and chemotaxis and are mitogens of various cell types. Fibrinogen alpha chain has also been identified as a diagnostic biomarker for cancers such as gastric cancer (Duan et al., 2018).

In this study, we aimed to explore the potential of the D-dimer-related genes F3, F5 and FGA as biomarkers for various cancer types. After using several bioinformatic tools to reveal D-dimer-related gene survival relationships, we focused on the immune functions of F3 and F5. The novel CIBERSORTx helped us link D-dimer-related genes with immune cell types. Our results demonstrated that D-dimer-related genes influence CD8^+^ T cells via TGF-beta signaling and have the potential to become cancer biomarkers, particularly for gastric cancer.

## Materials and Methods

### Data Acquisition

The RNA-seq data of 33 tumors were downloaded from The Cancer Genome Atlas (TCGA). TMB data, MSI data and clinical data, including age, sex, tumor grade, clinical stage, TNM stage, and survival time, were also downloaded. Gene Expression Omnibus (GEO) datasets of GSE84437, GSE78220, GSE67501 and IMvigor210 were also downloaded (Ascierto et al., 2016; Hugo et al., 2016; Yoon et al., 2020). Data extraction was performed using R software 4.1.0 (R Foundation for Statistical Computing, Vienna, Austria).

### TIMER2.0

TIMER2.0 is a comprehensive resource for systematic analysis of immune infiltrates across diverse cancer types (http://timer.cistrome.org). We used TIMER2.0 to analyze the differential expression (tumor vs. normal) in a pan-cancer prospective study (Li et al., 2017; Li et al., 2020).

### Gene Expression Profiling Interactive Analysis 2

Gene Expression Profiling Interactive Analysis 2 (GEPIA2) is an enhanced web server that conducts large-scale expression profiling and interactive analysis (http://gepia2.cancer-pku.cn) (Tang et al., 2017; Tang et al., 2019). Here, we used the webtool to generate Kaplan-Meier plots for D-dimer-related gene signatures.

### UALCAN

UALCAN is a web resource to analyze cancer OMICS data that is user-friendly, comprehensive and interactive (http://ualcan.path.uab.edu) (Chandrashekar et al., 2017). In this study, we used UALCAN to generate tumor vs. normal boxplots of D-dimer-related genes using TCGA STAD data.

### Kaplan-Meier Plotter

Kaplan-Meier Plotter is a webtool to assess the effect of 54k genes, as well as the mRNA, miRNA and protein, on survival in 21 cancer types, including gastric, lung, ovarian and breast cancer (www.kmplot.com) (Nagy et al., 2021). In the present study, we used Kaplan-Meier Plotter to generate Kaplan-Meier plots of D-dimer-related genes using gene chip data.

### Long-Term Outcome and Gene Expression Profiling Database of Pan-Cancers

Long-term Outcome and Gene Expression Profiling database of pan-cancers (LOGpc) included 209 expression datasets and 13 survival analyses of 27 different malignancies involving 31,310 patients (http://bioinfo.henu.edu.cn/DatabaseList.jsp) (Xie et al., 2020). We utilized LOGpc to generate survival plots using the gastric cancer data of GEO-GSE84437.

### Patients Specimens

Gastric cancer cDNA microarray from 30 GC patients (cDNA-HStmA060CS01), including 30 paired cancer and non-cancerous gastric tissues, were purchased from Shanghai Outdo Biotech Co., Ltd. (Shanghai, China). The access date of the microarray is 3.21.2017. This study was completed under the guidance of the International Ethical Guidelines for Biomedical Research Involving Human Subjects (CIOMS). The Clinical Research Ethics Committee of Shenyang First People's Hospital approved the research protocols. The characteristics of the GC patients’ data can be found in Table 1.

**TABLE 1:** Characteristics of GC patients used for qRT-PCR.

**Characteristics**	**Variables**	**Patients (30)**	**Percentages (%)**
Gender	Male	24	80
	Female	6	20
Age	<60	7	23.33
	≥60	23	76.67
T Stage	T1-2	4	13.33
	T3-4	26	86.67
N Stage	N0-1	16	53.33
	N2-3	14	46.67
Grade	1	2	6.67
	2–3	28	93.33
F3 2^(-△△CT)	<1	13	43.33
	≥1	17	56.67
F5 2^(-△△CT)	<1	12	40
	≥1	18	60

### Quantitative Reverse Transcriptase PCR

The cDNA used in this study was synthesized by RNA reverse transcription using Thermo Fisher’s SuperScript IV Reverse transcriptase (18090010). It was then used to detect the mRNA levels of F3, F5, and B-actin using Takara’s SYBR® Premix Ex Taq™ II (Tli RNaseH Plus) (RR820Q). The relative mRNA expression was determined using the cycle threshold (CT) formula 2^-△△CT^, where △CT = [CT (target gene)—CT (B-actin)]. The expression level was normalized against endogenous B-actin. The specific primers were as follows: human F3 forward 5′-GGA​ACC​CAA​ACC​CGT​CAA​TC-3′ and reverse 5′-AGG​AGA​AGA​CCC​GTG​CCA​AG-3′; human F5, forward 5′-TGA​CCT​TCT​CGC​CTT​ATG​A-3′ and reverse 5′-CTC​TGT​ATT​CCT​CGC​CTG​T-3'; human B-actin, forward 5′-GAA​GAG​CTA​CGA​GCT​GCC​TGA-3′ and reverse 5′- CAG​ACA​GCA​CTG​TGT​TGG​CG-3'.

### Venn Plots

Venn plots of this study were generated using an online tool (http://bioinformatics.psb.ugent.be/webtools/Venn/).

### Gene Ontology Enrichment Analysis

Gene Ontology (GO) enrichment analyses using 82 upregulated and 3 downregulated DEGs were performed using the R packages “clusterProfiler”3.16.1, “enrichplot”1.8.1, and “ggplot2”3.3.2. Terms with *p* < 0.05 were considered significant (Ashburner et al., 2000; Gene Ontology, 2021).

### Gene Set Enrichment Analysis

Gene set enrichment analysis (GSEA) was performed to reveal correlated pathways of D-dimer-related genes (Subramanian et al., 2005). Hallmark gene sets v7.2 were downloaded as the target sets, and GSEA was performed using GSEA_4.1.0 software. Gene sets with a nominal (NOM) *p* < 0.05 and a false discovery rate (FDR) q < 0.06 were considered significant.

### TICs Profile

The novel CIBERSORTx webtool was used to estimate the tumor-infiltrating immune cell (TIC) profile in tumor samples, and samples with *p* > 0.05 were eliminated from further study (Newman et al., 2015).

### Statistical Analyses

All statistical analyses were conducted using R software (version 4.1.0) and GraphPad Prism 9. The statistical significance threshold of all statistical tests was *p* < 0.05. The log rank test was used to evaluate the significance of Kaplan-Meier survival analysis.

## Results

### Identification of the Prognostic Value of D-Dimer-Related Genes in Gastric Cancer *via* a Pan-Cancer Study

To explore the value of the D-dimer-related genes F3, F5 and FGA, we conducted a pan-cancer study of these three genes and found a correlation among them and TMB, MSI and immune-related genes. According to the survival Kaplan-Meier plots grouped by the expression level of the three genes combined, we focused on a particular cancer type, gastric cancer, and explored the expression pattern within tumor and normal gastric samples along with their survival. F3 and F5, which showed higher expression in gastric cancer in qRT-PCR, were selected for further study. We conducted clinicopathology, gene set enrichment analysis and Gene Ontology-based studies and found that F3 and F5 affect TGF-beta signaling. Next, we performed immune cell analysis using CIBERSORTx and found a correlation between F3 and F5 with CD8^+^ T cells. The analytical workflow for the study is shown in Supplementary Figure S1.

### D-Dimer Related Genes Upregulated in Multiple Cancers

We first explored the differential expression of D-dimer-related genes between tumor and normal samples using TIMER2.0. The expression differentiation within the 33 cancer types was demonstrated via boxplots (Supplementary Figure S2). The names and abbreviations of these 33 cancer types can be found in the legend. The TIMER2.0 data suggested that F3 expression was significantly upregulated in CHOL and ESCA, F5 expression was significantly upregulated in BRCA, COAD, LIHC, LUAD, PAAD, PCPG, PRAD and STAD, and the expression of FGA was significantly upregulated in BLCA, COAD, HNSC and READ. Taken together, the results suggested that D-dimer-related genes are upregulated in multiple cancer types.

### D-Dimer-Related Gene Expression Is Correlated With TMB and MSI Levels

**FIGURE 1:**
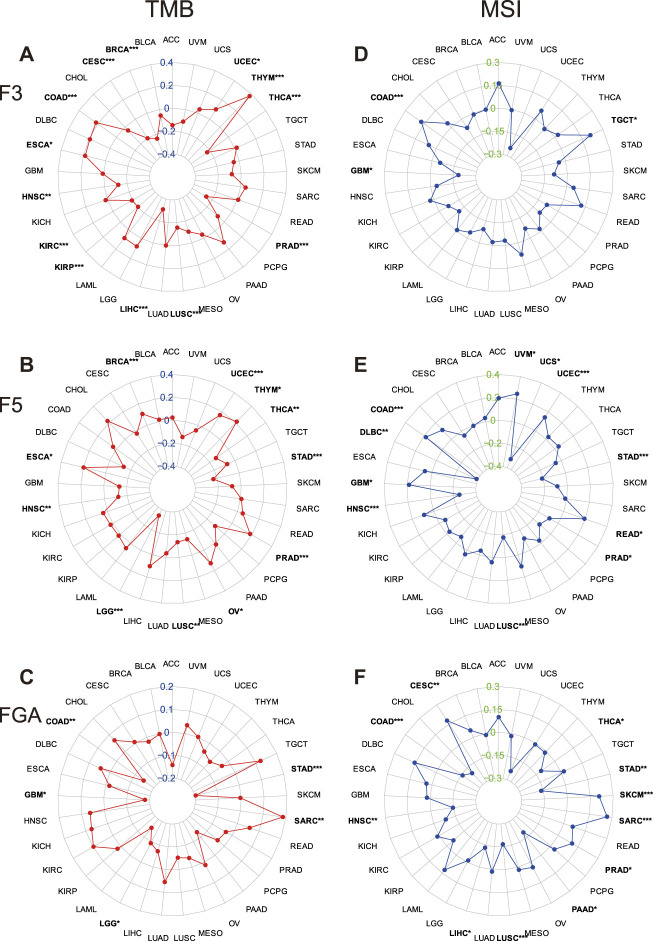
Correlation between the expression of D-dimer-related genes and the tumor mutation burden (TMB) and microsatellite instability (MSI) in TCGA cancers. **(A–C)** Correlation between the expression of D-dimer-related genes and TMB in 33 types of cancers. **(D–F)** Correlation between the expression of D-dimer-related genes and MSI in 33 types of cancers. * indicates *p* < 0.05, ** indicates *p* < 0.01, and *** indicates *p* < 0.001, every tumors with a significant p value was highlighted with a bold character.

Studies were conducted on the relationship between the gene expression levels and TMB and the MSI levels. TMB, or tumor mutation burden, represents the number of somatic mutations in the coding region of the tumor cell genome at an average size of 1 MB (Campbell et al., 2017). MSI, or microsatellite instability, refers to the random change in the microsatellite length and emergence of new microsatellite alleles in tumor cells because of the insertion or deletion of repeat units compared with normal cells (Cortes-Ciriano et al., 2017). Radar plots were drawn demonstrating the correlation between gene expression and TMB and MSI based on Spearman’s correlation (Figure 1). Significant correlations were found in multiple cancer types. F3 was most positively correlated with TMB in THYM and most negatively correlated with TMB in LIHC. F5 was most positively correlated with TMB in THYM, ESCA and PRAD and most negatively correlated with TMB in LGG. FGA was most positively correlated with TMB in SARC and most negatively correlated with TMB in STAD. F3 was most positively correlated with MSI in TGCTs and COAD and most negatively correlated with MSI in GBM. F5 was most positively correlated with MSI in UVM and most negatively correlated with MSI in DLBC. FGA was most positively correlated with MSI in SARC and most negatively correlated with MSI in PAAD.

### D-Dimer-Related Genes Display Correlations With Multiple Immune-Related Genes

**FIGURE 2:**
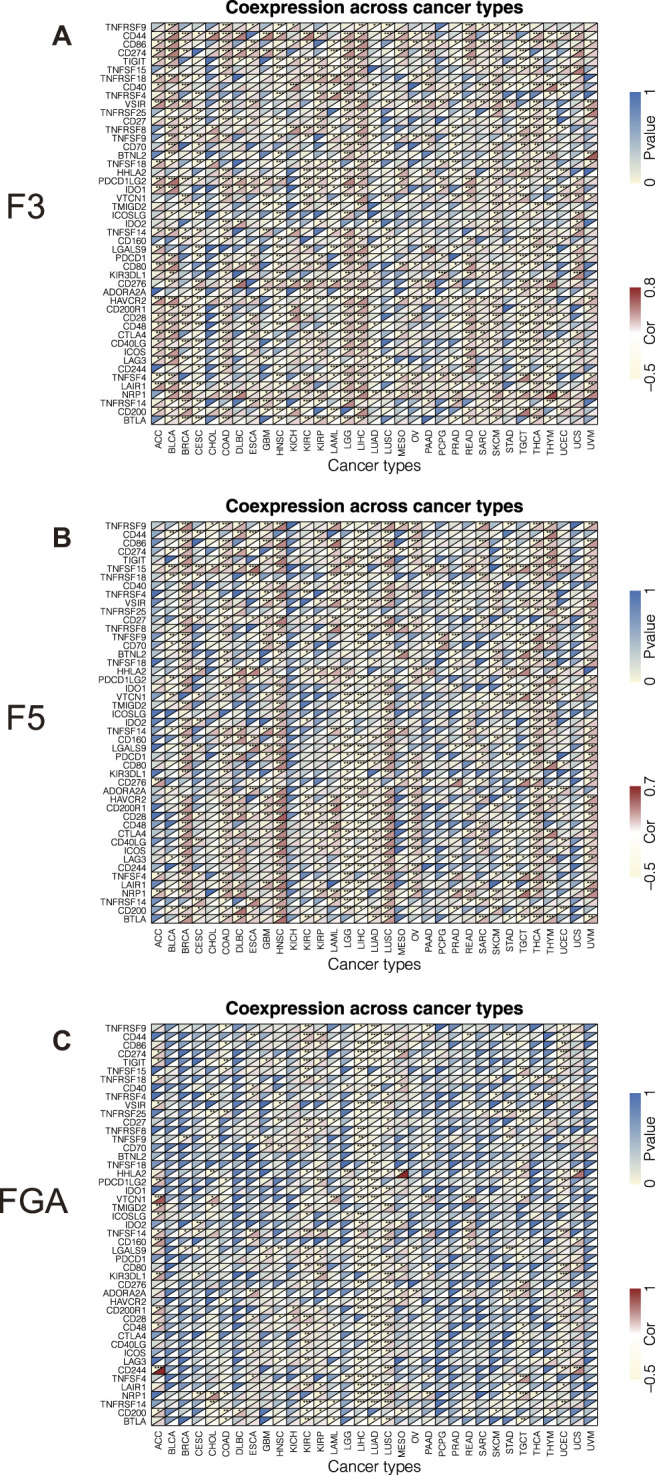
Correlation between the expression of D-dimer-related genes and immune-related genes in TCGA cancers. **(A–C)** Correlation of F3, F5 and FGA with 47 immune-related genes.

To explore the relationship between D-dimer-related genes and genes correlated with immunity, we drew heatmaps that displayed the correlation factor and P value of D-dimer genes with each immune-related gene (Figure 2). The heatmaps demonstrated that D-dimer-related genes had strong correlations with immune-related genes among multiple cancer types. F3 had correlations with 29 immune-related genes in LIHC, F5 had correlations with 28 immune-related genes in HNSC, and FGA had correlations with only 16 immune-related genes in LUAD. Comparing these three plots revealed that F3 and F5 are more immune correlated than FGA.

### D-Dimer-Related Genes Affect Patients’ Responsiveness to anti-PD1/PDL1 Therapy

**FIGURE 3:**
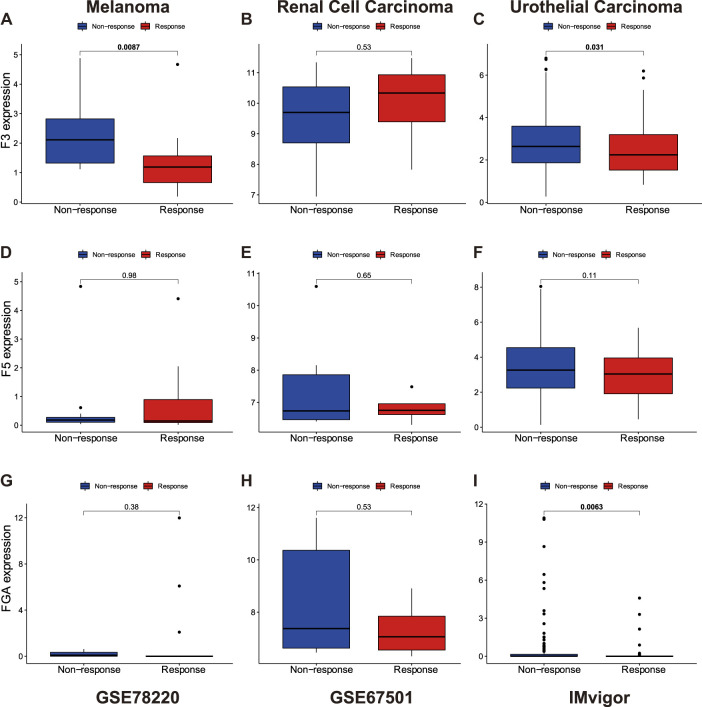
Responses of anti-PD1/PDL1 therapy are affect by D-dimer related genes expression. **(A–C)** The relationship between F3 expression and responsiveness of anti-PD1/PDL1 therapy in melanoma, renal cell carcinoma and urothelial carcinoma. **(D–F)** The relationship between F5 expression and responsiveness of anti-PD1/PDL1 therapy, similar with F3. **(G–I)** The relationship between FGA expression and responsiveness of anti-PD1/PDL1 therapy, similar with F3.

In order to explore whether D-dimer-related genes are capable of influencing the efficacy of anti-PD1/PDL1 therapy, we downloaded the responsiveness of anti-PD1/PDL1 therapy in three datasets, GSE78220 and GSE67501 of GEO, and also IMvigor210 dataset. The three datasets represent three types of cancer, namely melanoma, renal cell carcinoma and urothelial carcinoma. The transcriptome data included within the three datasets derived from pre-treatment tumors. We generated bar plots of the three genes, and we saw positive results in F3 (melanoma and urothelial carcinoma) and FGA (urothelial carcinoma) (Figure 3). The results are that D-dimer-related genes are potential influencers of anti-PD1/PDL1 therapy and suggest a possible link between D-dimer-related genes and CD8^+^ T cells.

### D-Dimer-Related Genes Are Correlated With Survival in Several Cancer Types

**FIGURE 4:**
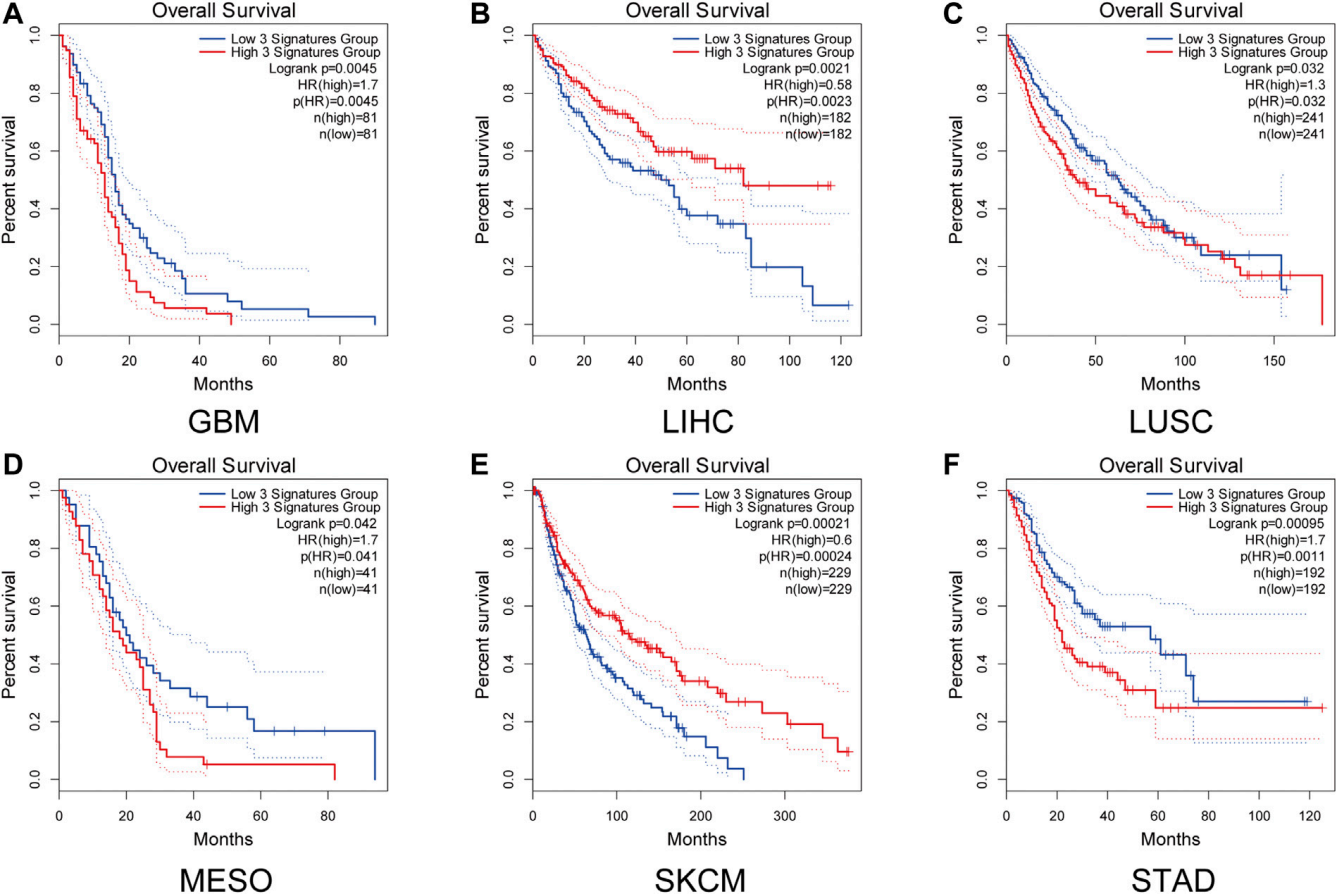
D-Dimer-related genes affect the survival of several types of tumors. **(A–F)** Kaplan-Meier plots of the three D-dimer-related genes in different tumor types using GEPIA2. *p* < 0.05 in GBM, LIHC, LUSC, MESO, SKCM AND STAD.

We next examined the survival correlations of D-dimer-related genes via Kaplan-Meier plots (Figure 4). We used GEPIA2 and explored the survival correlations by inputting the three genes as a gene set. High and low expression groups were determined by the medium of the expression of the three signature groups. The plots demonstrated that D-dimer-related gene signatures had survival correlations with six cancer types, GBM, LIHC, LUSC, MESO, SKCM and STAD. Among them, two had a hazard ratio smaller than 1, while four had a hazard ratio larger than 1. Among the four cancer types with a hazard ratio larger than 1, STAD had the smallest P value but the largest hazard ratio. The results suggested that the survival correlation of D-dimer-related genes was most significant within STAD or gastric cancer (GC) and that the combination of F3, F5 and FGA might be a potential multibiomarker for GC.

### D-Dimer-Related Genes Are Potential Clinicopathology-Related Biomarkers for Gastric Cancer

**FIGURE 5:**
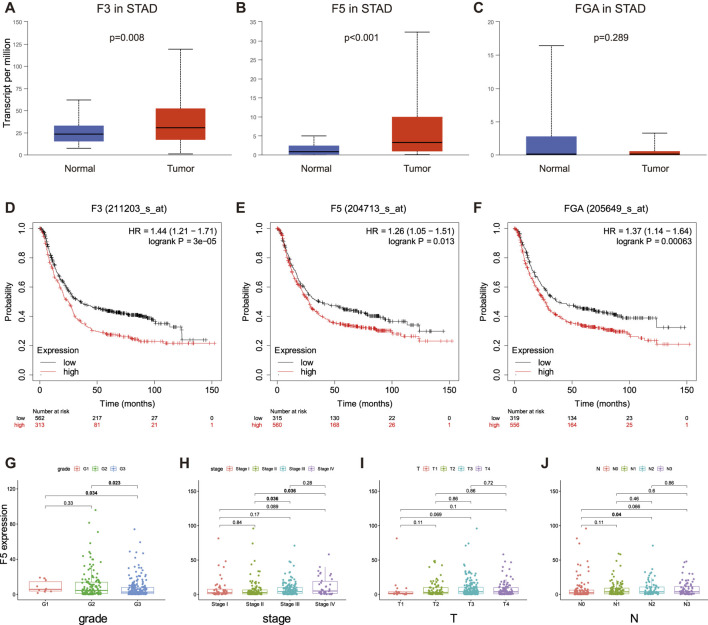
Survival correlation, differential expression and clinicopathological relations in D-dimer-related genes. **(A–C)** Expression levels of F3, F5 and FGA between GC and normal samples using UALCAN. **(D–F)** Kaplan-Meier plots of the three D-dimer-related genes in GC using KM plotter. **(G–J)** Relationship between F5 expression and clinicopathological parameters.

After focusing on STAD, we conducted studies on the differential expression and survival correlation using UALCAN and KM Plotter. A differential expression boxplot showed that F3 and F5 were overexpressed in tumor samples compared with normal samples, while FGA expression differentiation was not significant (Figures 5A–C). We then utilized KM Plotter to construct Kaplan-Meier plots with mRNA sequences. All three genes were correlated with survival in gastric cancer patients; the high D-dimer-related gene expression group had poorer survival than their low counterparts (Figures 5D–F). We also explored the relationship between D-dimer-related genes and the clinicopathological characteristics of GC patients. The four plots showed that F5, the most well-performed gene, had clear correlations with tumor grade, tumor stage, T and N (Figures 5G–J). The scatterplots of F3 and FGA were in Supplementary Figure S3. Taken together, the results suggested that D-dimer-related genes are potential clinicopathology-related GC biomarkers.

### Verification of D-Dimer-Related Genes’ Biomarker Potential in GC by qRT-PCR and GEO Cohort

**FIGURE 6:**
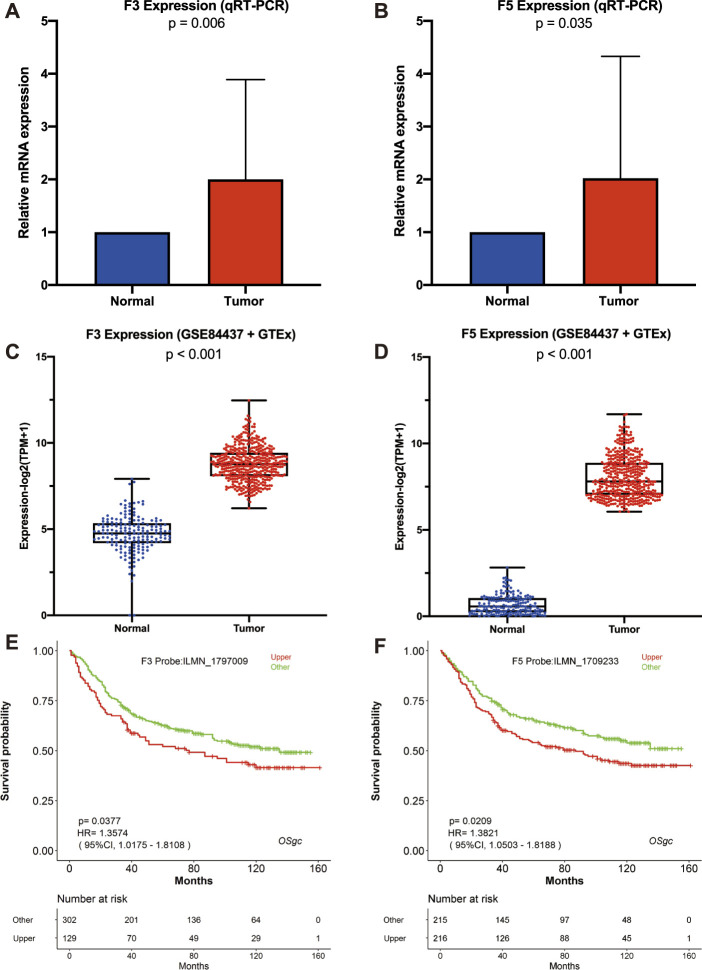
Validation of F3 and F5’s biomarker potential *via* qRT-PCR and GEO cohort. **(A–B)** F3 and F5 expression compared within normal and tumor tissues *via* qRT-PCR. **(C–D)** Comparison of F3 and F5 expression *vi*a GEO-GSE84437 and GTEx data. **(E–F)** Kaplan-Meier plots of F3 and F5 using GSE84437 data, generated *via* LOGpc.

In hope of verifying D-dimer-related genes F3 and F5’s upregulation in GC and their survival correlation, we first conducted quantitative real-time polymerase chain reaction on the two genes. Patients’ data, including clinicopathological data and 2^-△△CT^ data, can be found in Table 1, while raw data of 2^-△△CT^ can be found in Supplementary Table S1 **.** Demonstrated in Figures 6A,B, the relative mRNA expression of both F3 and F5 were discovered to upregulate in tumor tissues comparing with adjacent normal tissues. The result of the validation experiment was in coordinate with our in-silico exploration result, the expression fold change of each sample can be found in Supplementary Figure S4. We then utilized a GEO gastric cancer cohort, namely GSE84437. The expression difference test was conducted, and the result also proves our former speculation (Figures 6C,D). We also generated Kaplan-Meier plots using GSE84437 data. The results of which was similar with our previous KM test, suggesting higher F3 and F5 expression to link with worse prognosis (Figures 6E,F). The results of our validation tests were supportive to our previous results and improved the reliability of F3 and F5’s biomarker potential.

### Gene Set Enrichment Analysis Reveals the Functions of D-Dimer Related Genes

**FIGURE 7:**
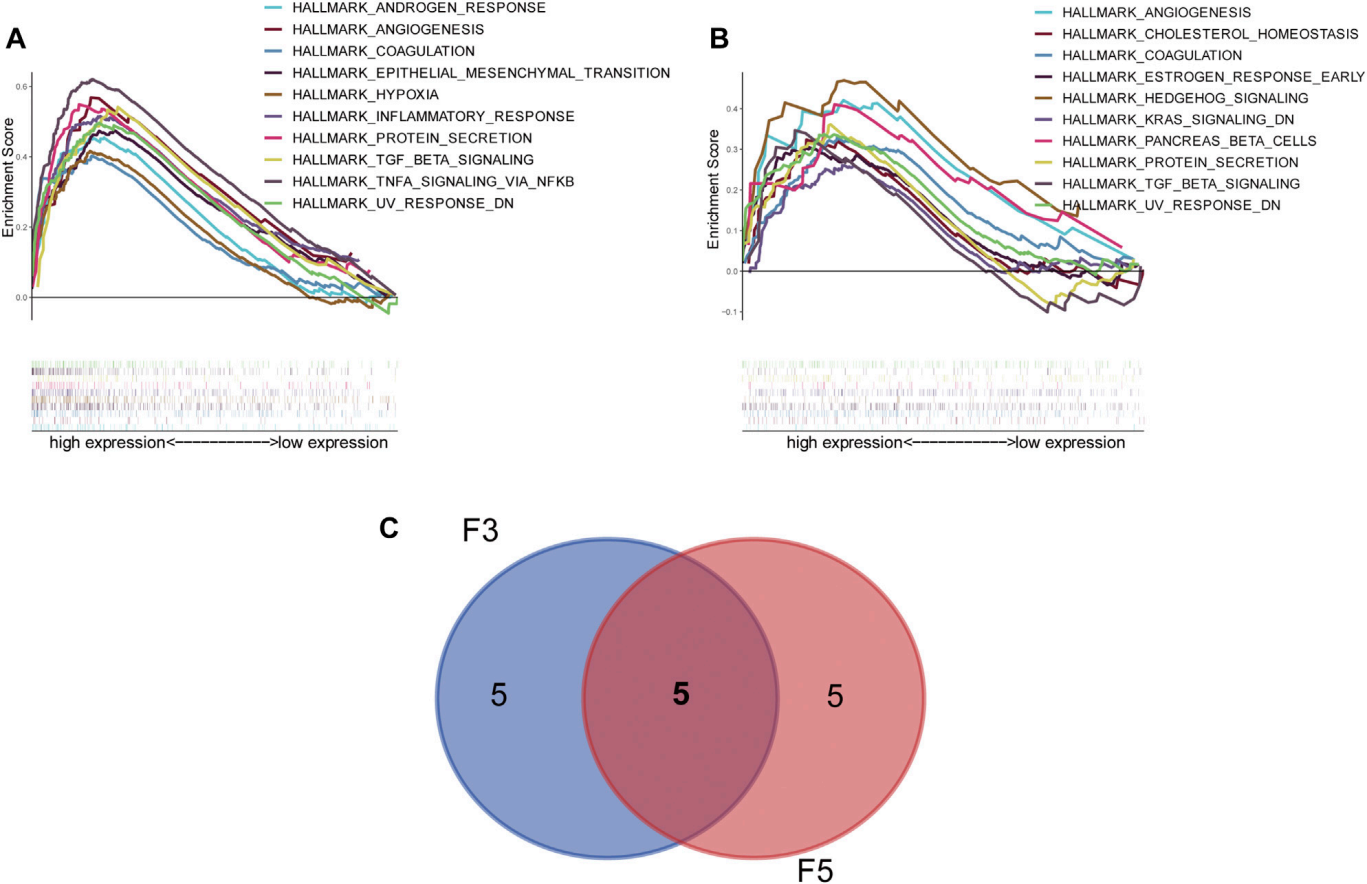
Gene set enrichment analysis (GSEA) reveals the correlation of the D-dimer-related gene expression with several HALLMARK pathways. **(A)** Multi-GSEA constructed within F3 (high *vs.* low). **(B)** Multi-GSEA constructed within F5 (high *vs*. low). **(C)** Venn plot displaying the overlapping pathways of F3 and F5—namely, angiogenesis, coagulation, protein secretion, TGF beta signaling and UV response down.

To explore the functions of D-dimer-related genes, we performed gene set enrichment analysis (GSEA) of both F3 and F5. The multi-GSEA plots are shown in Figures 7A,B, gene set data for GSEA can be found in Table 2; Supplementary Table S2. Pathways such as coagulation, which is consistent with the apparent functions of coagulation factors, were upregulated in both the F3 and F5 high expression groups. A Venn plot was drawn to show the overlapping pathways of F3 and F5 (Figure 7C). In total, five pathways overlapped—angiogenesis, coagulation, protein secretion, TGF-beta signaling and UV response down. Although the upregulation of angiogenesis and coagulation suggested the regular functions of coagulation factors, the upregulation of TGF-beta signaling might suggest that our two genes have potential functions within the immune system. In summary, the upregulated pathways suggested that F3 and F5 might be related to tumor growth and metastasis.

**TABLE 2:** GSEA HALLMARK gene set data for F3.

**F3 Gene set name**	**NES**	**NOM p-value**	**FDR q-value**
HALLMARK_TNFA_SIGNALING_VIA_NFKB	2.1254597	0.0020920502	0.012768714
HALLMARK_PROTEIN_SECRETION	1.8423452	0.011538462	0.108587116
HALLMARK_TGF_BETA_SIGNALING	1.8364073	0.004201681	0.07587484
HALLMARK_ANDROGEN_RESPONSE	1.8044238	0.003992016	0.07571529
HALLMARK_INFLAMMATORY_RESPONSE	1.7778409	0.025210084	0.07510248
HALLMARK_ANGIOGENESIS	1.7481711	0.03218884	0.0744541
HALLMARK_UV_RESPONSE_DN	1.7308366	0.023809524	0.07183651
HALLMARK_HYPOXIA	1.7283051	0.006147541	0.063461505
HALLMARK_KRAS_SIGNALING_UP	1.5838146	0.04140787	0.14405042
HALLMARK_ESTROGEN_RESPONSE_EARLY	1.5765619	0.01192843	0.13539882
HALLMARK_COAGULATION	1.5047035	0.07083333	0.18064147
HALLMARK_P53_PATHWAY	1.4900132	0.064327486	0.17695041
HALLMARK_EPITHELIAL_MESENCHYMAL_TRANSITION	1.4614903	0.14529915	0.1858001
HALLMARK_IL2_STAT5_SIGNALING	1.3936588	0.0931677	0.23351207
HALLMARK_COMPLEMENT	1.3718619	0.1211499	0.23838237
HALLMARK_APOPTOSIS	1.3674948	0.09543569	0.22706622
HALLMARK_APICAL_JUNCTION	1.339846	0.124740124	0.23955816
HALLMARK_ESTROGEN_RESPONSE_LATE	1.3308438	0.08139535	0.23490565
HALLMARK_NOTCH_SIGNALING	1.303096	0.12	0.24633111
HALLMARK_CHOLESTEROL_HOMEOSTASIS	1.2884008	0.19881889	0.24660629
HALLMARK_IL6_JAK_STAT3_SIGNALING	1.2409605	0.24032587	0.27643096
HALLMARK_HEME_METABOLISM	1.16959	0.21	0.3391025
HALLMARK_APICAL_SURFACE	1.0963137	0.32291666	0.41219345
HALLMARK_XENOBIOTIC_METABOLISM	0.9730441	0.48765433	0.5603313
HALLMARK_BILE_ACID_METABOLISM	0.97177833	0.47843942	0.53951836
HALLMARK_GLYCOLYSIS	0.95500576	0.5	0.5430764
HALLMARK_MITOTIC_SPINDLE	0.89950985	0.5173745	0.60172385
HALLMARK_HEDGEHOG_SIGNALING	0.798115	0.7014028	0.7355321
HALLMARK_KRAS_SIGNALING_DN	0.61187136	0.9920477	0.9415714
HALLMARK_INTERFERON_GAMMA_RESPONSE	0.5211765	0.8945233	0.9624734

### Differential Expression Analysis Identifies Functions of D-Dimer Related Genes in Metabolic Processes

**FIGURE 8:**
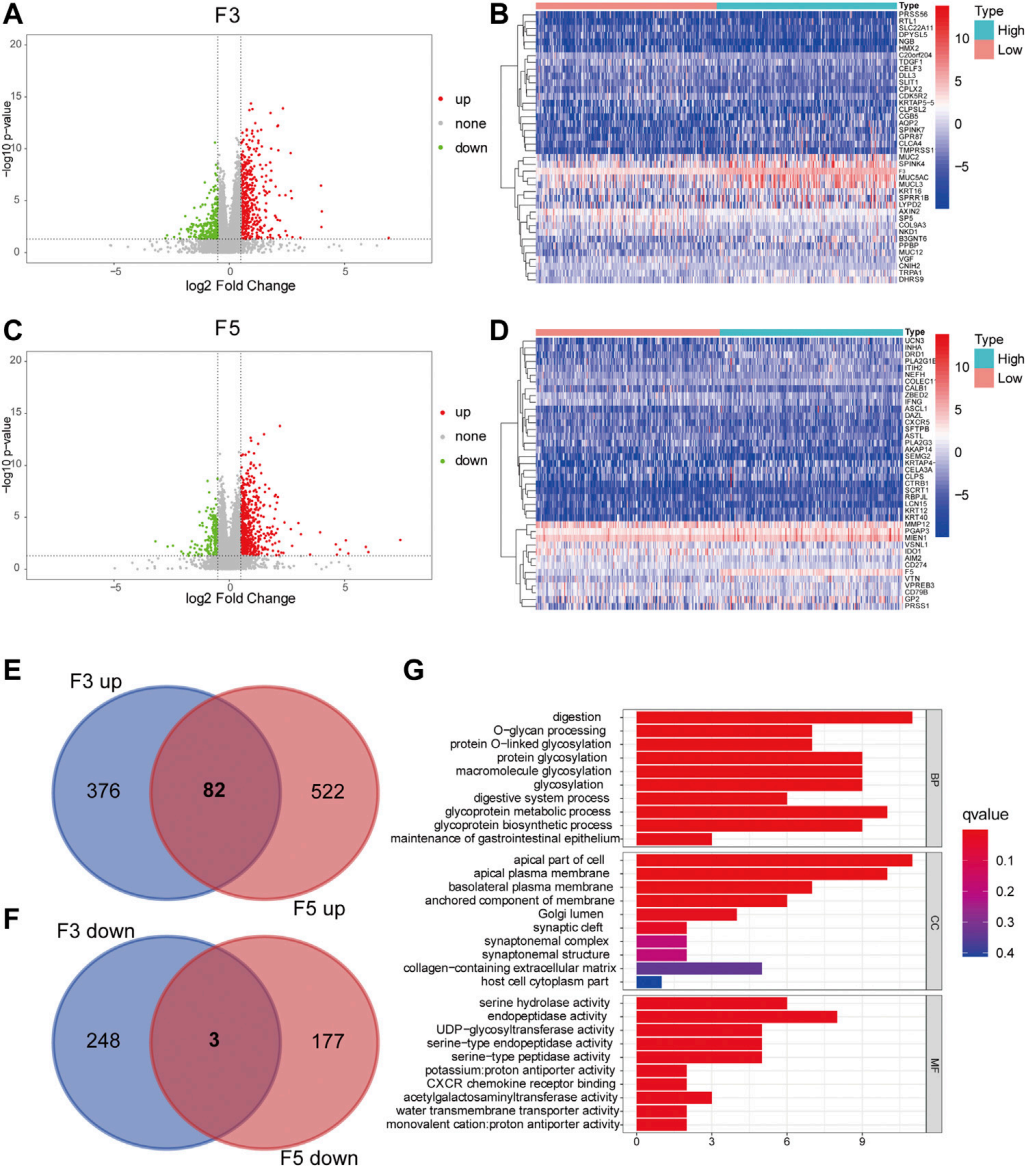
GO enrichment of overlapping DEGs. **(A)** Volcano plot of the DEGs of F3 (high and low). *p* < 0.05 was the cutoff criterion for DEG significance, and an absolute value of log_2_-fold change>0.5 was considered significant. The red dots represent genes that are upregulated, the green dots represent genes that are downregulated, and the black dots resemble genes that are nonsignificant. **(B)** Heatmap depicting the top 20 upregulated and downregulated genes with F3 high and low expression. A higher fold change is depicted with a redder color. **(C)** Volcano plot of the DEGs of F3 (high and low expression), similar to **(A)**. **(D)** Heatmap depicting the top 20 upregulated and downregulated genes with F5 high and low expression, similar to **(B)**. **(E–F)** Venn plot displaying the overlapping genes of F3 and F5. **(G)** GO plot illustrating different enriched pathways of overlapping DEGs.

To identify genes that are expressed correlatively with D-dimer-related genes, we conducted differential expression analysis between the high and low F3 and F5 expression groups. Volcano plots and heatmaps were constructed (Figures 8A–D), and Venn plots demonstrated the overlapping genes of F3 and F5 (Figures 8E,F). In total, 82 genes were upregulated in both the F3 and F5 comparison groups, while only 3 genes were downregulated consistently. Gene Ontology was conducted on these 85 genes, and we observed upregulated functions in both biological processes (BP), cellular components (CC) and molecular functions (MF), including glycoprotein metabolic process and apical part of cell (Figure 8G). Thus, genes related to F3 and F5 had functions in the tumor metabolic process.

### D-Dimer Related Genes Are Potential Influencers of TICs

**FIGURE 9:**
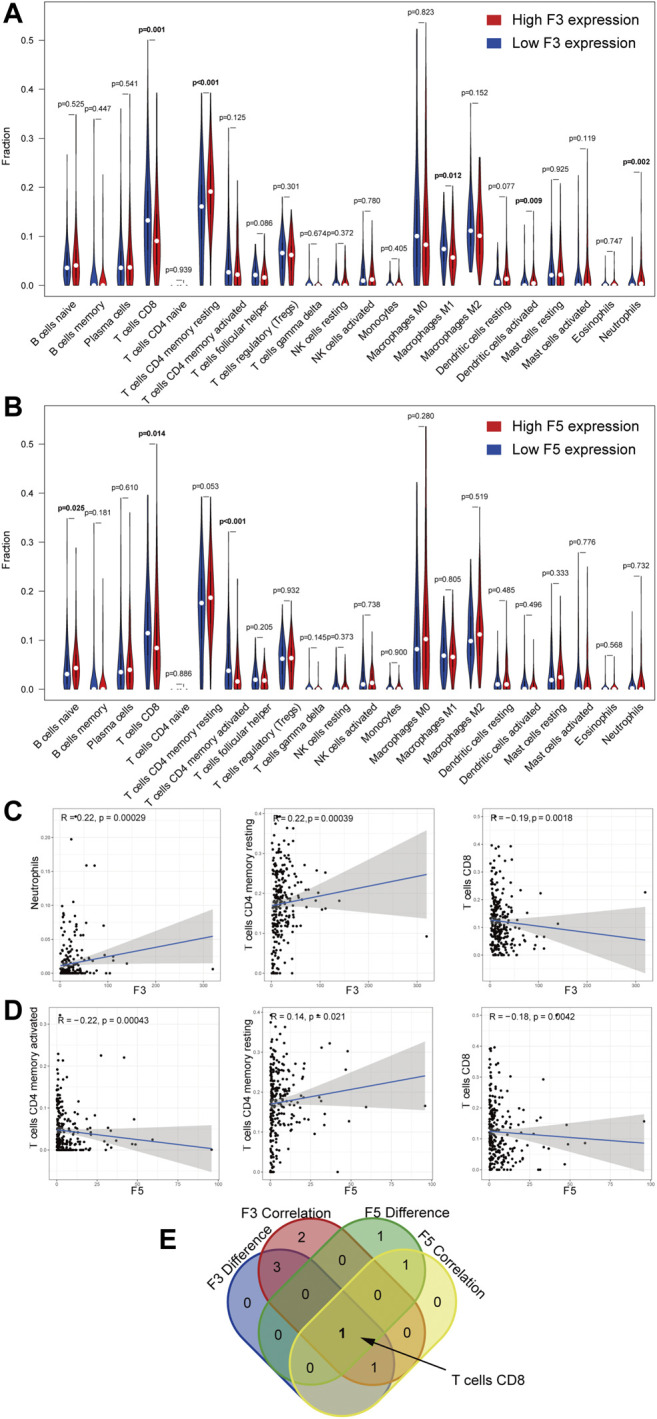
Correlation of the TIC proportion with the expression of D-dimer-related genes. **(A–B)** Violin plot displaying the ratio differentiation of 22 immune cell types between STAD tumor samples with low or high F3 and F5 expression relative to the median gene expression level. Wilcoxon rank sum for the significance test. **(C–D)** Scatter plots showing the correlation of the top 3 TIC proportions with F3 and F5 expression (*p* < 0.05). Pearson’s coefficient for the correlation test. **(E)** Venn plot displaying the overlapping F3 and F5 cells—namely, CD8 T cells.

After we explored the immune relationship of D-dimer-related genes with immunity via immune gene correlation, we analyzed the proportion of tumor-infiltrating immune cells using the novel CIBERSORTx webtool. The STAD immune cell profiles are shown in Supplementary Figure S5. Next, we independently constructed the difference test and correlation test of immune cells using both F3 and F5 (Figures 9A–D). After an overlap demonstration using a Venn plot, we found that CD8^+^ T cells overlapped in all four tests, including the difference test and correlation test of F3 and the difference test and correlation test of F5 (Figure 9E). Additional verification using xCell method suggests similar results (Supplementary Figure S3). These results suggest that CD8-positive T cells can be influenced by the expression of D-dimer-related genes.

## Discussion

D-Dimer is a soluble fibrin degradation product produced by the orderly decomposition of thrombi by fibrinolytic systems. Many studies have shown that D-dimer is a vital marker of coagulation and fibrinolytic activity. Therefore, D-dimer has been widely used to diagnose venous thromboembolism (VTE). Because cancer patients are at a higher risk of developing VTE, VTE has become a crucial influencer of the prognosis of cancer patients. Previous studies have concluded that VTE is the second leading cause of death in cancer patients (Blom et al., 2006). Thus, surveillance of VTE for cancer patients will most likely benefit these patients. Previous studies have found that thrombocythemia is common in gastrointestinal cancer, ovarian cancer, lung cancer and breast cancer, possibly reducing the threshold required for VTE (Hisada and Mackman, 2017). Thus, the D-dimer level, one of the most reproducible predictors of thromboembolism, should be monitored in patients with cancers such as gastric cancer. The biomarker function of D-dimer inspired us to examine its capabilities in more depth, thus encouraging us to conduct related genetic studies.

In the present study, we first evaluated D-dimer-related genes using a pan-cancer method. We found that the D-dimer-related genes F3, F5 and FGA were differentially expressed in cancer tissues and normal tissues in multiple cancer types, such as STAD and PAAD. The expression differentiation between cancer and normal tissues enabled the three D-dimer-related genes to be used as cancer biomarkers. Tissue factor and factor V, the proteins encoded by F3 and F5, were proved by previous studies to be linked with cancer-associated thrombosis and survival of cancer patients (Thaler et al., 2012; Crous-Bou et al., 2016; Rondon et al., 2019). Our findings further proved F3 and F5’s potential of influencing VTE of cancer patients in a molecular level.

The tumor mutation burden (TMB) and microsatellite instability (MSI) are important factors to classify cancer phenotypes and predict the survival of cancer patients. F3 had the most positive correlation with TMB in THYM and the most negative correlation with TMB in LIHC. F5 had the most positive correlation with TMB in THYM, ESCA and PRAD and the most negative correlation with TMB in LGG. FGA had the most positive correlation with TMB in SARC and the most negative correlation with TMB in STAD. F3 had the most positive correlation with MSI in TGCT and COAD and the most negative correlation with MSI in GBM. F5 had the most positive correlation with MSI in UVM and the most negative correlation with MSI in DLBC. FGA had the most positive correlation with MSI in SARC and the most negative correlation with MSI in PAAD. Our results suggest that D-dimer-related genes are related to TMB and MSI.

Anti-PD1/PDL1 therapy is by far one of the most well-known immunotherapies for cancer treatment (Han et al., 2020). By blocking the PD1/PDL1 checkpoint, cancer cell loses its capability of evading from the attacks of T cells (Jin et al., 2011; Tumeh et al., 2014). Nonetheless, it was discovered that some patients failed to respond to anti-PD1/PDL1 therapy (Nowicki et al., 2018). In our study, we utilized three datasets that provided data on the responses of anti-PD1/PDL1 therapy. We discovered that non-response patients tend to express higher D-dimer related genes. In hope of providing an explanation of this phenomenon, we looked through previous studies and found reports on the potential mechanism that expression of D-dimer related gene to be influenced by T infiltrating lymphocytes and, in turn, influences VTE (Gi et al., 2021). In which case, D-dimer related gene could potentially reflect T infiltration level, therefore being capable of suggesting the responsiveness of anti-PD1/PDL1 therapy.

Prognostic biomarkers have been used to predict the survival of cancer patients in recent years. Although biomarkers such as CA125 are successful for survival prediction, most single biomarkers have failed to provide optimal testing results for cancer patients (Dochez et al., 2019). Previous studies have claimed that the concept of a “single marker” in cancer is incorrect because several pathways and processes in tumor cells have changed (Rodríguez-Enríquez et al., 2011). Thus, identifying novel multibiomarkers is necessary. In our study, we tested the prognostic value of the three D-dimer-related genes F3, F5 and FGA as multibiomarkers in 33 cancer types. Kaplan-Meier plots suggested that elevations in D-dimer-related gene expression in GBM, LUSC, MESO and STAD were prognostic, with STAD being the most satisfactory in both hazard ratios and statistical significance. Our study demonstrated that the combination uses of F3, F5 and FGA as multibiomarkers is prognostic in several cancer types, most ideally in gastric cancer.

Considering that the prognostic value of D-dimer-related genes is most satisfactory in gastric cancer, we assessed the independent prognostic value of F3, F5 and FGA. Although all three genes exhibited survival correlation via Kaplan-Meier plots, only F3 and F5 showed expression differentiation between tumor and normal tissues in STAD. The results ruled out the possibility of FGA being a satisfactory independent GC biomarker. Independent GEO cohort and qRT-PCR results verified F3 and F5’s biomarker potential. Further exploration into the correlation between D-dimer-related gene expression and GC clinicopathological characteristics showed that D-dimer-related gene F3 and F5 expression is correlated with GC clinicopathological characteristics such as N (nodal metastasis).

Previously developed bioinformatic methods such as Gene Set Enrichment Analysis (GSEA) and Gene Ontology (GO) have allowed scientists to explore the functions of selected gene sets. These analytical methods advanced the studies of multiple diseases, including cancer. Our team first used GSEA to examine the related functions of F3 and F5. Consistent with our common knowledge, the coagulation pathway is upregulated in both the F3 and F5 gene sets. Additionally, other pathways that were found to be upregulated enabled the identification of these D-dimer-related genes. Pathways that are commonly regarded as cancer-related were found among the upregulated pathways, such as hypoxia (Jing et al., 2019). TGF-beta signaling, which is also well recognized as a crucial signaling pathway for cancer development, was influenced in both the F3 and F5 gene sets (Katz et al., 2013). After conducting differential expression analysis between the high and low F3 and F5 expression groups, we obtained the overlapping genes of F3 and F5. Gene Ontology was conducted on these 85 genes, and upregulated functions were observed in BP, CC and MF, including glycoprotein metabolic process and apical part of cell, a finding that is consistent with our upregulated pathway of protein secretion in GSEA. The functions of genes related to F3 and F5 potentially influence the tumor metabolic process.

CIBERSORT, or cell-type identification by estimating relative subsets of RNA transcripts, is an analytical tool that uses gene expression data to estimate the abundance of member cell types in a mixed cell population. This tool allowed exploration of the linkage of selected gene expression with immune cell fractions. Because GSEA revealed potential upregulation of immune-related pathways of D-dimer-related genes, we focused on immune functions. First, we conducted a correlation test between the three D-dimer-related genes and immune-related genes using a pan-cancer method. The results imply that F3 and F5 are more immune correlated than FGA. Analysis of previously found immune checkpoints, such as PD-L1 and CTLA4, revealed that both F3 and F5 were positively correlated with CD274 (PD-L1) in LIHC and MESO; both F3 and F5 were positively correlated with CTLA4 in COAD, GBM, LGG, LIHC, READ, SARC, TGCT and THCA. These findings suggest that F3 and F5 are well immunocorrelated with cancers such as LIHC. Inspired by previous reports of the potential mechanism that expression of F3 to be influenced by T infiltrating lymphocytes and, in turn, influences VTE, we propose that genes related to T cell infiltration (CD274, CTLA4, etc.) might be risk factors of VTE. Additional correlation tests were conducted between F3 and F5 and immune cell markers of CD8^+^ T cells and macrophages within STAD, which are immune cell types that we previously studied (Wu et al., 2020; Guan et al., 2021). We found that F5 was negatively correlated with CD8A, which is a marker of CD8^+^ T cells. By facilitating CIBERSORTx, a novel version of CIBERSORT, we linked the F3 and F5 expression levels with the immune cell fraction. Overlap of the correlation test and difference test of F3 and F5 revealed that both F3 and F5 are negatively correlated with the cell fraction of CD8^+^ T cells in STAD, a finding that is consistent with our result with the CD8^+^ T cell marker CD8A. Our study demonstrated that D-dimer-related genes are associated with CD8^+^ T cells. Combined with our discovery of TGF-beta signaling upregulation via GSEA, we could link the decrease in the CD8^+^ T cell fraction with the upregulation of the TGF-beta signaling pathway. Consistent with previous studies showing that neutralizing TGF-beta enhances the antitumor immune response mediated by CD8^+^ T cells, we concluded that F3 and F5 expression levels influence the TGF-beta signaling pathway and influence the enrichment of CD8^+^ T cells, demonstrating the immune function of F3 and F5 in an F3,F5/TGF-beta signaling/CD8+ T cell axis (Yang et al., 2010). Our study successfully identified a new function for D-dimer-related genes.

## Conclusion

Our group conducted a pan-cancer study of three D-dimer-related genes—F3, F5 and FGA—and revealed their potential as multibiomarkers as well as single markers for cancer, particularly gastric cancer. Additional studies were conducted on the functions of D-dimer-related genes, and upregulated pathways, such as coagulation and protein secretion, were identified. Immune-related characteristics were explored, and F3 and F5 were identified as potential inhibitors of CD8^+^ T cells. Our results demonstrated that the three D-dimer-related genes are potential GC multibiomarkers that correlate with TMB and MSI and have immune-related functions, regulating CD8^+^ T cells. Further study of D-dimer-related genes will help improve the survival of cancers such as GC.

## Data Availability

The datasets presented in this study can be found in online repositories. The names of the repository/repositories and accession number(s) can be found in the article/Supplementary Material.
