# Supplementary material for: Pan-Cancer Analysis and Validation Reveals that D-Dimer-Related Genes are Prognostic and Downregulate CD8+ T Cells via TGF-Beta Signaling in Gastric Cancer
Source: Front Mol Biosci. 2022 Feb 22;9:790706. doi: 10.3389/fmolb.2022.790706 (PMC8902139; doi:10.3389/fmolb.2022.790706)
Supplement: Supplementary file 3 [file DataSheet2.DOCX]

**Pan-cancer Analysis Revealed the Prognosis Value of D-Dimer Related Genes within Gastric Cancer**

SUPPLEMENTARY MATERIALS


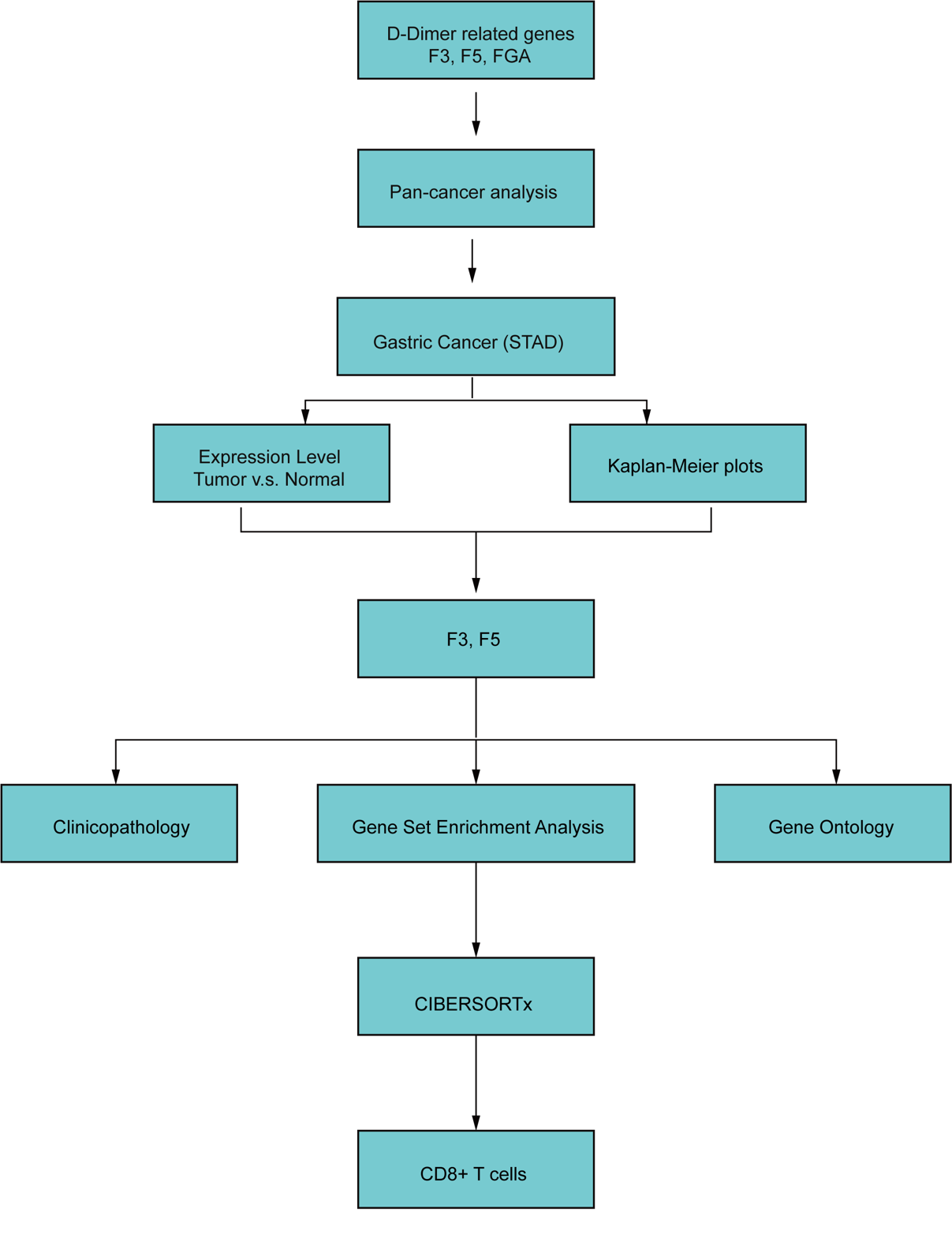
**Figure S1.** Workflow of the present research.

**
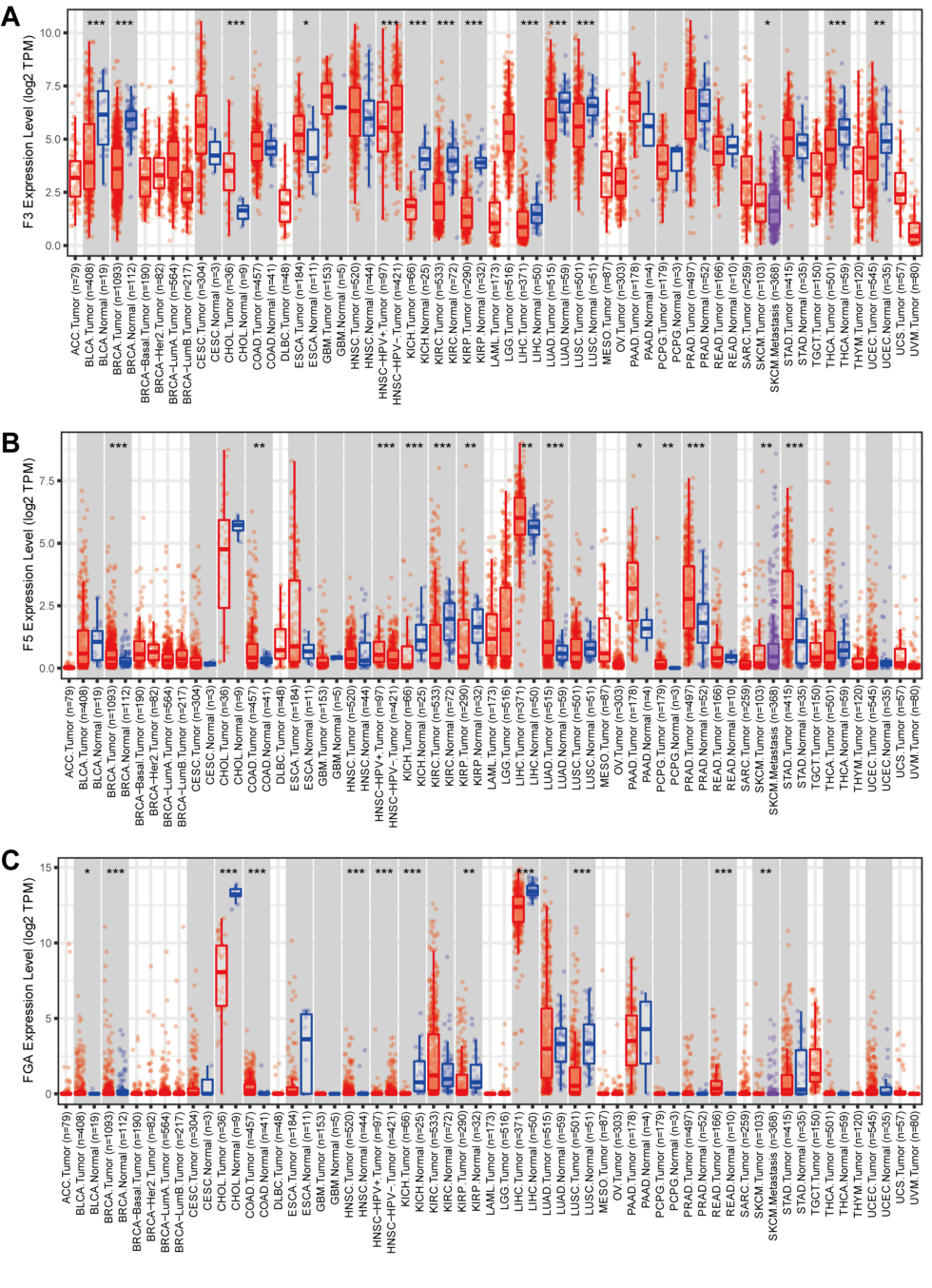
**

**Figure S2.** D-Dimer-related gene expression varies in different tumor types. **(A-C)** Expression levels of F3 **(A)**, F5 **(B)** and FGA **(C)** in multiple tumor types and normal samples using TIMER. Abbreviation: Adrenocortical carcinoma (ACC), Bladder Urothelial Carcinoma (BLCA), Breast invasive carcinoma(BRCA), Cervical squamous cell carcinoma and endocervical adenocarcinoma (CESC), Cholangiocarcinoma (CHOL), Colon adenocarcinoma (COAD), Lymphoid Neoplasm Diffuse Large B-cell Lymphoma (DLBC), Esophageal carcinoma (ESCA), Glioblastoma multiforme (GBM), Head and Neck squamous cell carcinoma (HNSC), Kidney Chromophobe (KICH), Kidney renal clear cell carcinoma (KIRC), Kidney renal papillary cell carcinoma (KIRP), Acute Myeloid Leukemia (LAML), Brain Lower Grade Glioma (LGG), Liver hepatocellular carcinoma (LIHC), Lung adenocarcinoma (LUAD), Lung squamous cell carcinoma (LUSC), Mesothelioma (MESO), Ovarian serous cystadenocarcinoma (OV), Pancreatic adenocarcinoma (PAAD), Pheochromocytoma and Paraganglioma (PCPG), Prostate adenocarcinoma (PRAD), Rectum adenocarcinoma (READ), Sarcoma (SARC), Skin Cutaneous Melanoma (SKCM), Stomach adenocarcinoma (STAD), Testicular Germ Cell Tumors (TGCT), Thyroid carcinoma(THCA), Thymoma (THYM), Uterine Corpus Endometrial Carcinoma (UCEC), Uterine Carcinosarcoma (UCS) and Uveal Melanoma (UVM).


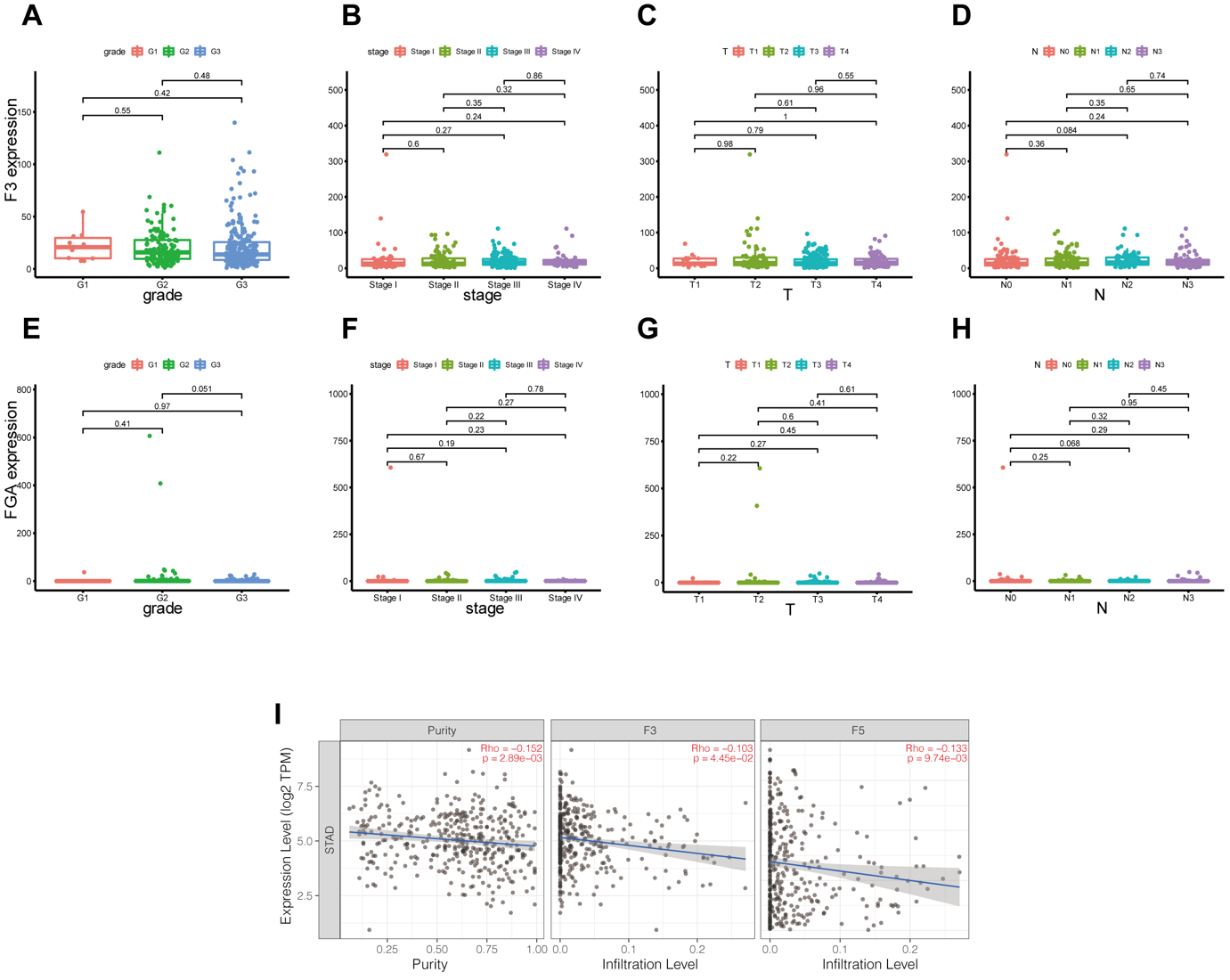


**Figure S3.** Correlation between F3, FGA expression with clinicopathology, and between F3, F5 expression and CD8+ T cells infiltration level.


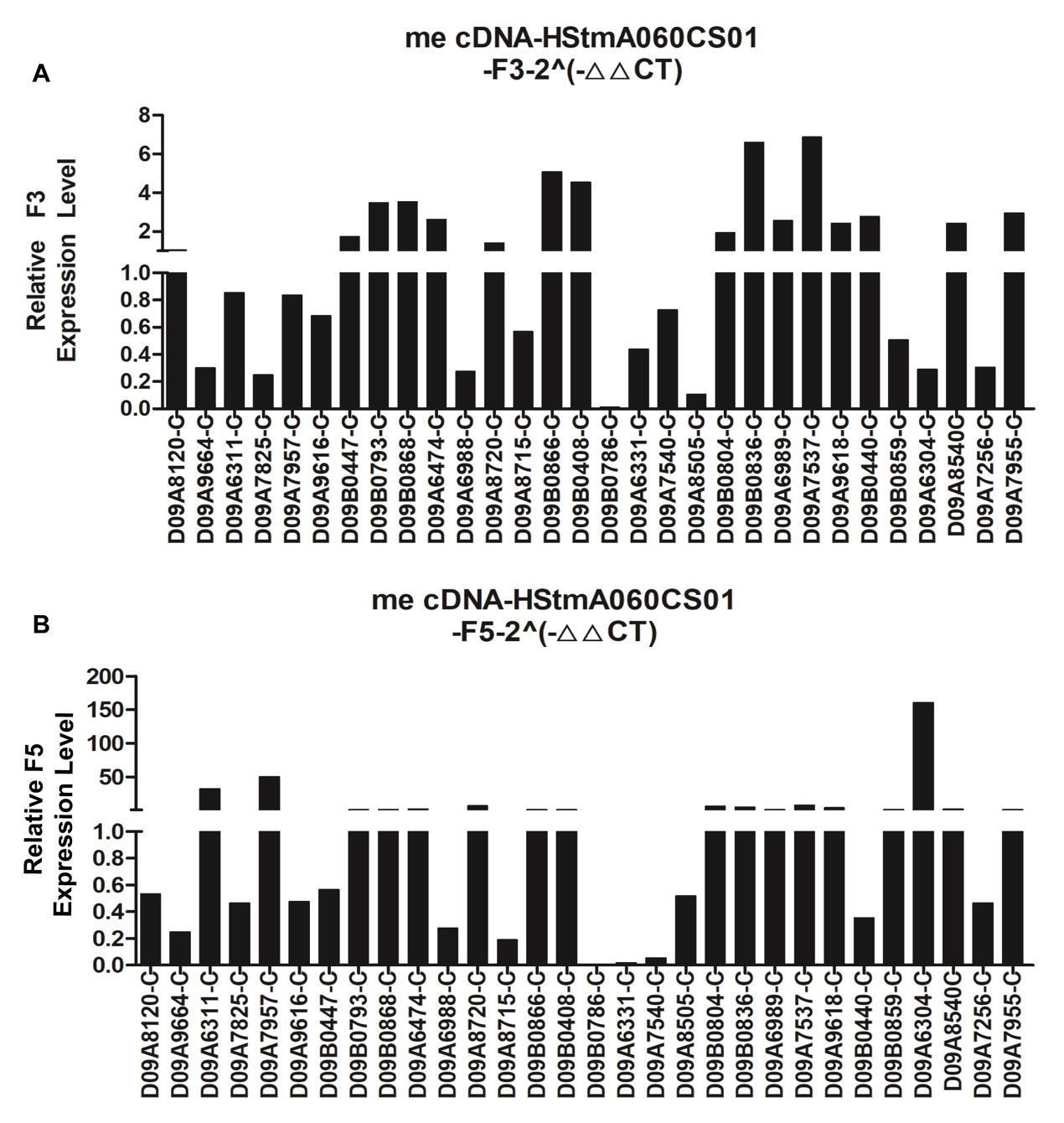


**Figure S4.** qRT-PCR results of F3 and F5, demonstrating the expression difference of tumor and adjacent normal tissues. 2^- ΔΔ CT^ indicates the differential multiple of the expression of this gene in cancer and adjacent tissues. A number lower than 1 indicates that the expression is low in cancer, the expression is high in cancer if the number is larger than 1.


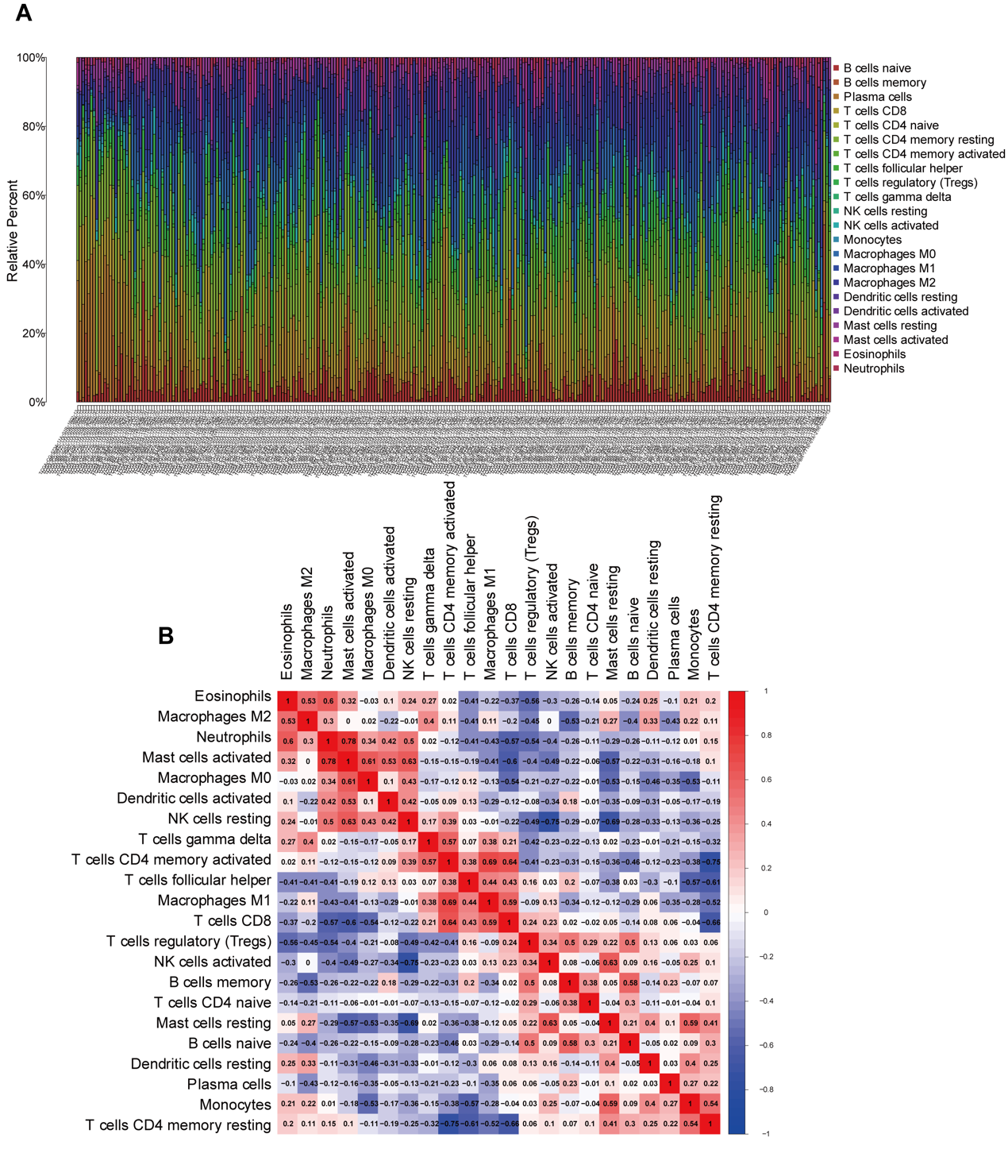


**Figure S5.** CIBERSORTx-derived TICs profile and correlation analysis. **(A)** Barplot displaying the proportion of 22 kinds of TICs within TCGA STAD samples. Sample ID were the column names. **(B)** Heatmap showing the correlation between 22 kinds of TICs. Correlation coefficient between cells were inside each box. A higher correlation was represented with a redder shade color. Pearson coefficient for significance test.
