# Supplementary material for: Pan-Cancer Analysis and Validation Reveals that D-Dimer-Related Genes are Prognostic and Downregulate CD8+ T Cells via TGF-Beta Signaling in Gastric Cancer
Source: Front Mol Biosci. 2022 Feb 22;9:790706. doi: 10.3389/fmolb.2022.790706 (PMC8902139; doi:10.3389/fmolb.2022.790706)
Supplement: Supplementary file 4 [file DataSheet1.DOCX]

Table 1. Characteristics of GC patients used for qRT-PCR.

Table 2. GSEA HALLMARK gene set data for F3.

Supplementary Table 1. Raw data of 2^(-△△CT) for F3 and F5.

Supplementary Table 2. GSEA HALLMARK gene set data for F5.
